# South African Flag Sign in Acute Myocardial Infarction: A Case Report and Literature Review Highlighting the Superiority of the Occlusion Myocardial Infarction (OMI)/Non-Occlusion Myocardial Infarction (NOMI) Paradigm

**DOI:** 10.7759/cureus.96287

**Published:** 2025-11-07

**Authors:** Tamer Zahdeh

**Affiliations:** 1 Internal Medicine, Montefiore St. Luke's Cornwall, Newburgh, USA

**Keywords:** acute coronary syndrome, d1 occlusion, electrocardiography (ecg), high lateral myocardial infarction, occlusion myocardial infarction, omi/nomi paradigm, percutaneous coronary intervention, south african flag sign, stemi-equivalent, stemi/nstemi paradigm

## Abstract

The South African flag sign (SAFS) is a recently described electrocardiographic (ECG) pattern characterized by ST-segment elevation in leads I, aVL, and V2, with reciprocal ST-segment depression in lead III. It represents a subtle but specific marker of acute occlusion of the first diagonal (D1) branch of the left anterior descending (LAD) artery and is considered a ST-elevation myocardial infarction (STEMI)-equivalent pattern within the occlusion myocardial infarction (OMI) paradigm. We report the case of a 69-year-old male who presented with chest pain and fatigue. Initial ECG demonstrated normal sinus rhythm with deep Q waves in leads I and aVL, minimal ST-segment depression in the inferior leads, and subtle ST-segment elevation in leads I, aVL, and V2 - findings retrospectively consistent with the SAFS. Cardiac biomarkers were markedly elevated, and echocardiography revealed anterolateral wall hypokinesis with preserved left ventricular systolic function. Coronary angiography identified a 100% thrombotic occlusion of the D1 artery and a 90% complex stenotic lesion extending from the proximal to mid-LAD. Percutaneous coronary intervention (PCI) with drug-eluting stent placement was successfully performed in both lesions, restoring flow. The patient was discharged on dual antiplatelet therapy and high-intensity statin. This case highlights the importance of detailed ECG interpretation for early recognition of STEMI-equivalent patterns such as the SAFS, a subtle but reliable indicator of acute diagonal artery occlusion, and underscores the clinical relevance of the OMI/non-occlusion myocardial infarction (NOMI) paradigm, which more accurately identifies acute vessel occlusion than the traditional STEMI/non-ST-elevation myocardial infarction (NSTEMI) framework.

## Introduction

Electrocardiography (ECG) has long been the cornerstone diagnostic tool in acute coronary syndrome (ACS), dictating which patients receive emergent reperfusion. For decades, clinical practice has been governed by the ST-elevation myocardial infarction (STEMI) versus non-ST-elevation myocardial infarction (NSTEMI) dichotomy. This framework, however, rests on the assumption that ST-segment elevation is a reliable surrogate for acute coronary occlusion (ACO). Robust evidence now challenges this assumption, showing that only 40%-43% of occlusion myocardial infarction (OMI) cases fulfill STEMI criteria, while the remainder risk misclassification as NSTEMI, leading to delays in reperfusion and worse outcomes [1,2].

Against this backdrop, the South African flag sign (SAFS) has emerged as a distinctive ECG finding first described in educational cardiology forums and increasingly recognized in the peer-reviewed literature. Dr. László Littmann is credited with coining the term “South African Flag Sign” as a mnemonic teaching tool to help clinicians identify high-lateral infarction involving noncontiguous leads (I, aVL, and V2) with reciprocal ST depression in inferior leads. The pattern derives its name from its visual resemblance to the Y-shaped geometry and central stripe of the South African national flag when the corresponding ECG leads are superimposed on a 4 × 3 lead layout [3].

While the underlying principle - recognizing ischemic vectors projecting toward I/aVL and noncontiguous precordial leads - has earlier conceptual roots, the formal “flag” analogy is relatively recent. In 2016, Littmann published an influential teaching article titled "South African Flag Sign: A Teaching Tool for Easier ECG Recognition of High Lateral Infarct," which formally introduced the term and proposed its use to improve recognition and reduce missed D1 occlusions [3]. A year earlier, Durant and Singh (2015) had described a nearly identical ECG pattern of ST elevation in I, aVL, and V2 with inferior ST depression in a patient with acute first diagonal artery (D1) occlusion, now regarded as the clinical substrate upon which Littmann’s mnemonic was built [4]. In subsequent years, multiple case reports have expanded the SAFS concept to variant presentations, occasionally involving non-D1 occlusions [5].

The classic SAFS is defined by ST-segment elevation in leads I, aVL, and V2, with reciprocal ST-segment depression in lead III (and often aVF). Pathophysiologically, occlusion of the first diagonal branch of the left anterior descending (LAD) artery leads to ischemia directed toward a vector between 0° and −60° in the frontal plane (favoring I and aVL) and toward V2 in the horizontal plane. Consequently, reciprocal changes appear in the inferior leads (III/aVF) [3]. When viewed on a standard 12-lead ECG grid in a 3 × 4 orientation, this pattern of ST deviations matches the green Y-shaped stripe of the South African flag, hence the mnemonic. Because V2 is not contiguous with I/aVL, the SAFS often escapes traditional STEMI algorithms, which rely on contiguous lead groupings, and may therefore be misinterpreted as nonspecific ST changes or labeled as lateral ischemia.

Recognition of the SAFS carries immediate clinical urgency. As with any OMI, delayed reperfusion increases infarct size, mechanical complications, and mortality. Therefore, recognition of SAFS should prompt immediate activation of an emergent invasive strategy with priority for percutaneous coronary intervention (PCI), similar to conventional STEMI pathways. Data show that NSTEMI patients with angiographically proven OMI have nearly twice the mortality of those without occlusion, driven primarily by treatment delay rather than inherent pathophysiologic differences [1]. In a real-world registry of 334 ACS patients, 40% of OMI did not meet STEMI criteria; these STEMI(-) OMI patients experienced substantial delays to catheterization, yet their need for PCI and complication rates were virtually identical to those of STEMI(+) OMI [1]. This emphasizes why recognition of SAFS and other subtle STEMI-equivalent ECG patterns is essential, as delayed intervention carries outcomes comparable to missed occlusive events.

The SAFS epitomizes the STEMI(-) OMI problem. Under the traditional STEMI/NSTEMI model, such cases - particularly when ST-segment deviations are subtle - are often labeled NSTEMI despite representing true occlusions requiring immediate reperfusion. In contrast, the OMI/non-occlusion myocardial infarction (NOMI) paradigm classifies infarctions by the presence (OMI) or absence (NOMI) of acute occlusion, regardless of whether STEMI criteria are met [1,6]. Blinded validation studies have shown that expert ECG interpretation guided by OMI criteria doubles the sensitivity for detecting acute occlusion compared with STEMI thresholds (86% vs. 41%) while maintaining comparable specificity (2). These findings align with American College of Cardiology consensus statements warning that reliance on STEMI criteria alone will inevitably miss a significant proportion of patients with ACO [6].

The transition from STEMI/NSTEMI to OMI/NOMI represents a conceptual evolution akin to the earlier shift from Q-wave/non-Q-wave myocardial infarction (MI) classifications. As McLaren et al. noted, medical paradigms evolve until accumulating anomalies, such as missed OMIs, demand scientific redefinition. The OMI framework addresses these anomalies by integrating subtle ECG signatures (SAFS, hyperacute T waves, de Winter T waves, Wellens’ syndrome, posterior MI patterns, Aslanger pattern, and Sgarbossa criteria) with biomarkers and imaging to identify occlusions more accurately. The SAFS, in particular, has been consistently cited as a hallmark OMI pattern associated with diagonal branch occlusion, reinforcing its diagnostic and educational importance [6].

In this case report, we present a patient whose initial ECG demonstrated the SAFS, later confirmed by angiography to represent a total D1 occlusion. Through this case and accompanying literature review, we highlight the diagnostic importance of SAFS, its place within the OMI/NOMI paradigm, and the need for broader adoption of this framework to improve recognition and outcomes in ACO.

## Case presentation

A 69-year-old White male with the past medical history of babesiosis and Lyme disease presented with complaints of chest pain and fatigue. The patient reported that during the week prior to admission, he had been physically overexerting himself at home. Two days before admission, he had sudden-onset, crushing, sternal chest pain at rest, radiating to the upper back, along with a dull, aching discomfort in the left shoulder and arm. The episode lasted approximately 20 minutes and was not associated with diaphoresis, palpitations, syncope, or identifiable triggering, relieving, or exacerbating factors. On the following day, he woke up feeling profoundly weak and was unable to get out of bed, which prompted his presentation to the emergency department (ED). The patient noted that he had experienced intermittent, unprovoked episodes of chest pain over the past two years but denied difficulty breathing or chest discomfort while ambulating or climbing stairs. He denied smoking tobacco, vaping, or using illicit drugs but endorsed daily alcohol intake of approximately 1.5 ounces of rye or bourbon. Family history was notable for his elder brother undergoing PCI with stent placement when he was 45 years old.

**Figure 1:**
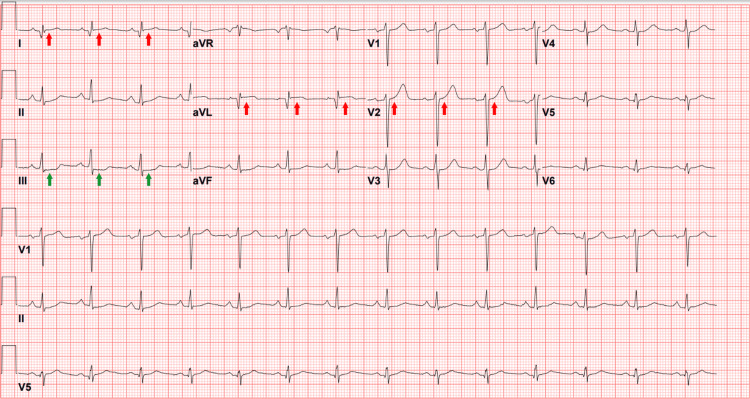
The patient's ECG showing normal sinus rhythm with subtle ST-segment elevation in leads I, aVL, and V2 (red arrows), and reciprocal ST-segment depression in lead III (green arrows), forming the South African flag sign (SAFS)

In the ED, the patient was hemodynamically stable, showed no acute respiratory distress, and had vital signs within normal limits on room air. Initial electrocardiography (ECG) was interpreted by the attending cardiologist as showing normal sinus rhythm with normal axis, normal PR/QT intervals, narrow QRS complex, deep Q waves in leads I and aVL, and minimal ST-segment depression in the inferior leads (Figure 1). Chest radiography was unrevealing for cardiomegaly or pulmonary vascular congestion. Blood workup was remarkable for evidence of acute cardiac injury with markedly elevated cardiac biomarkers consistent with myocardial infarction (Table 1). The patient was administered a loading dose of aspirin and started on intravenous unfractionated heparin infusion, then admitted with a working diagnosis of NSTEMI and plans for left heart catheterization (LHC) the following morning.

**Table 1:** Initial laboratory results on presentation Laboratory reference ranges are provided for comparison and correspond to adult male values.

Laboratory Test	Result	Reference Range	Units
White blood cell count (WBC)	11.0 × 10³	4.0–10.0 × 10³	/µL
Aspartate aminotransferase (AST)	117	10–40	U/L
Creatine phosphokinase (CPK)	660	30–200	U/L
Creatine kinase-MB fraction (CK-MB)	28	<5	ng/mL
High-sensitivity troponin I (initial)	26,000	<14	ng/L
High-sensitivity troponin I (repeat)	20,000	<14	ng/L
B-type natriuretic peptide (BNP)	2,700	<100	pg/mL
Serum creatinine	1.1	0.6–1.3	mg/dL
Blood urea nitrogen (BUN)	17	7–20	mg/dL
Sodium	138	135–145	mmol/L
Potassium	4.1	3.5–5.0	mmol/L
Glucose	106	70–110	mg/dL

Transthoracic echocardiography on the following morning showed normal left ventricular (LV) size, asymmetric interventricular septal hypertrophy, anterolateral wall hypokinesis, low-normal LV systolic function with an estimated ejection fraction of 50%-55%, a moderately dilated left atrium, normal right atrium and right ventricular size and function, structurally normal cardiac valves, and a normal-sized inferior vena cava with preserved collapsibility. There was no evidence of pleural effusion or pulmonary hypertension.

**Figure 2:**
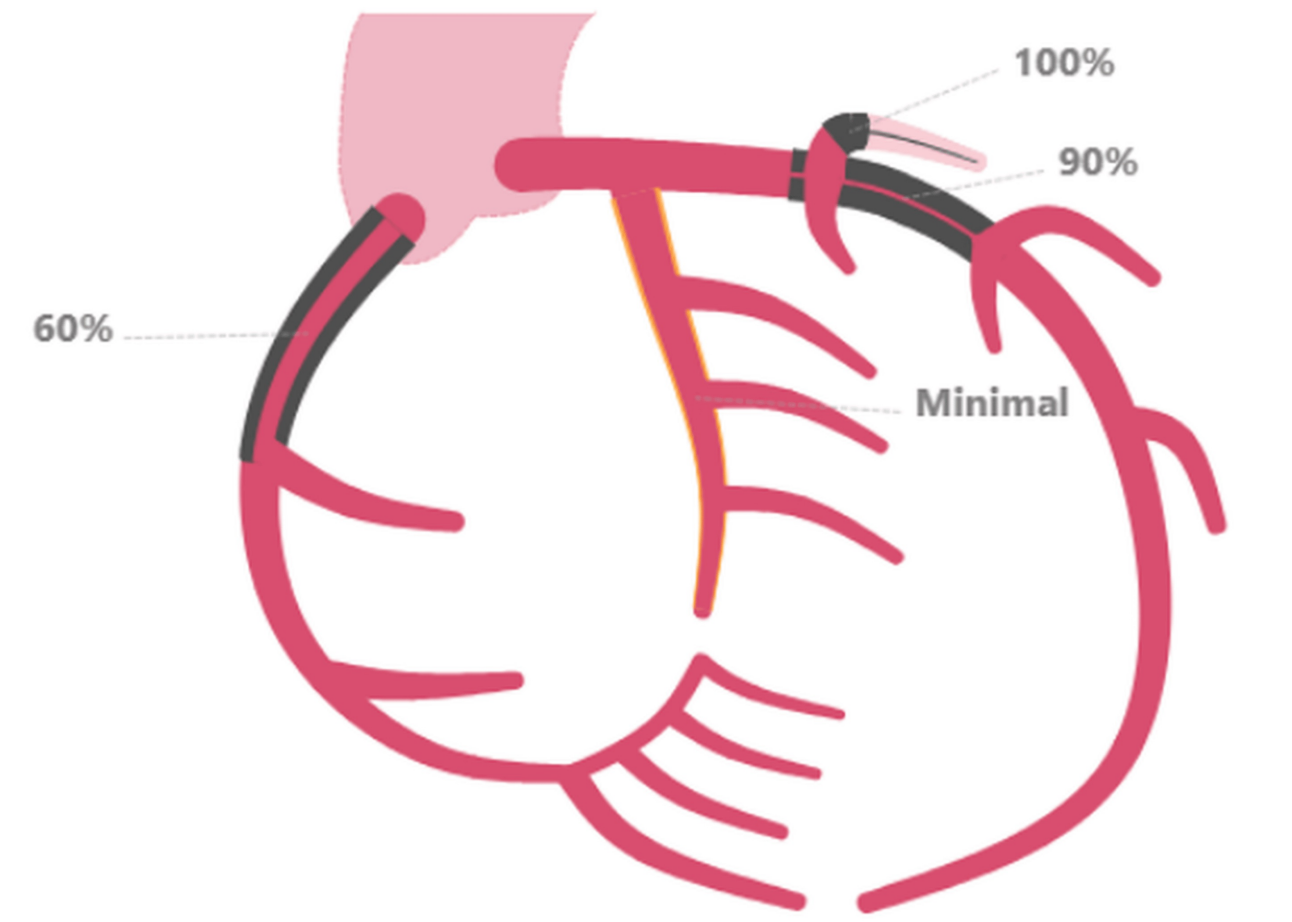
Schematic representation of the patient's coronary anatomy demonstrating 100% thrombotic occlusion of the first diagonal (D1) branch, 90% stenosis of the proximal to mid-left anterior descending (LAD) artery, and 60% stenosis of the proximal right coronary artery (RCA)

**Figure 3:**
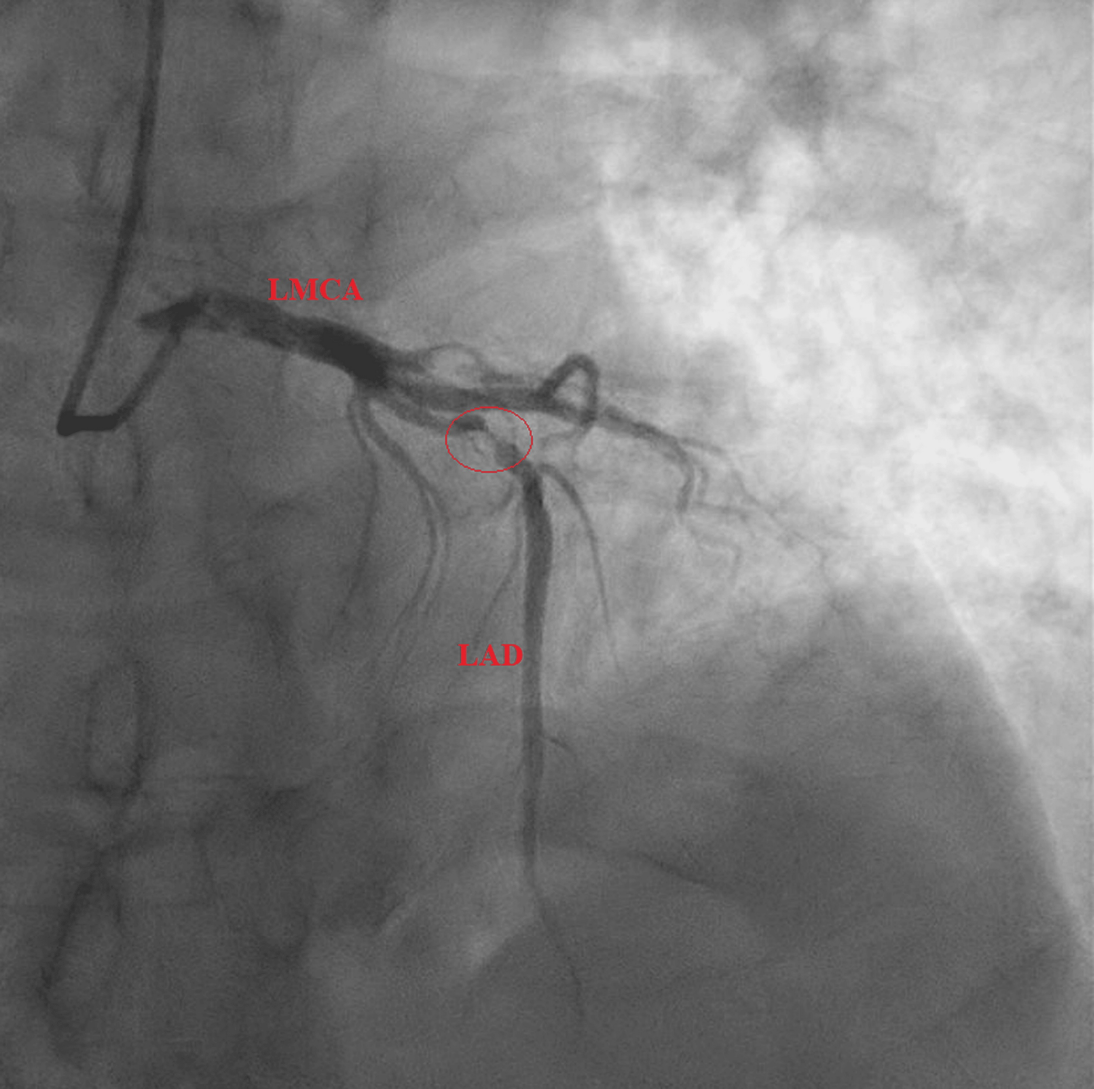
Right anterior oblique (RAO) cranial view of the left coronary system prior to percutaneous coronary intervention showing a high-grade stenosis in the proximal left anterior descending (LAD) artery (red circle)

**Figure 4:**
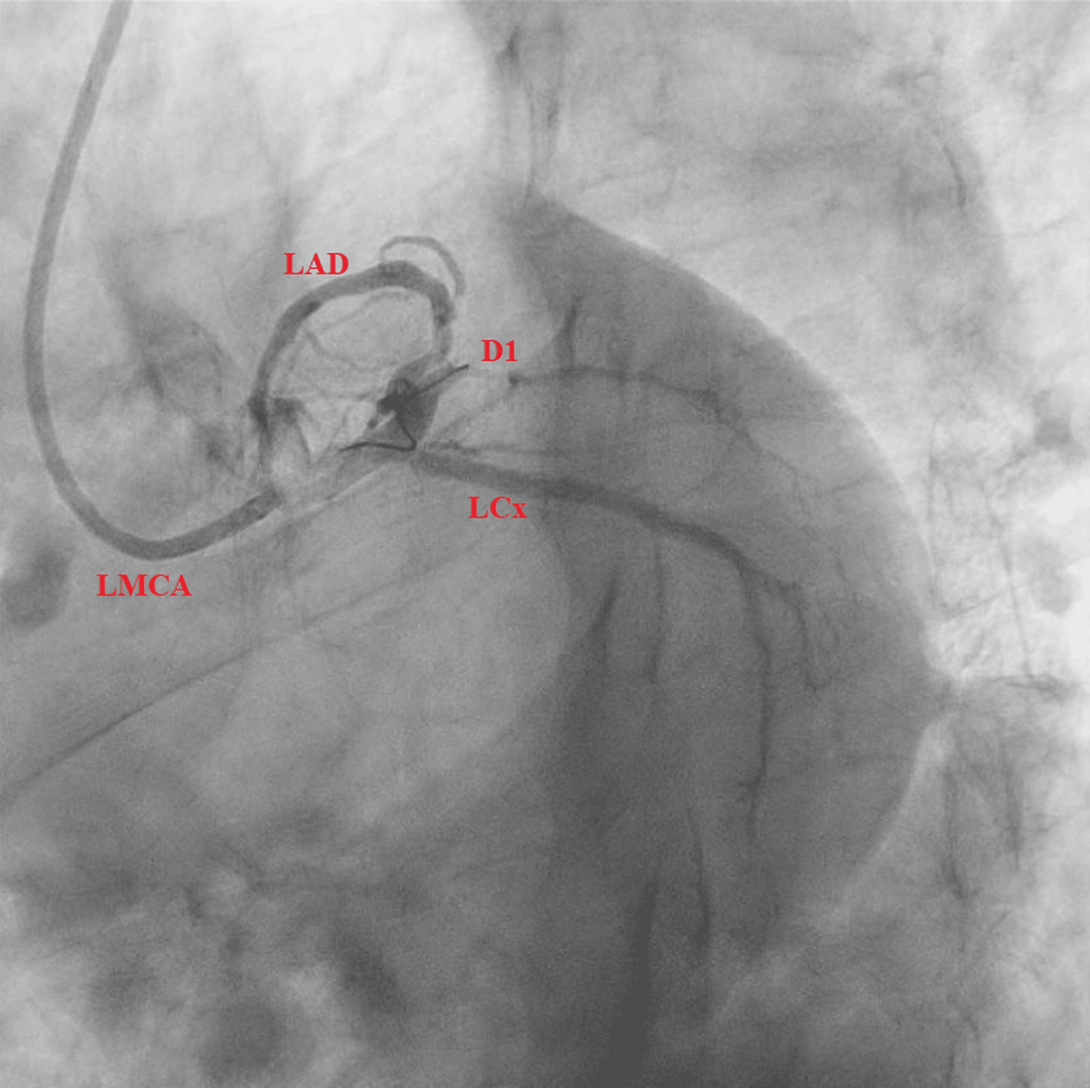
Caudal view showing a short left main coronary artery (LMCA) bifurcating into the left anterior descending (LAD) and left circumflex (LCx) arteries. The first diagonal (D1) branch arising from the proximal LAD is totally occluded.

Subsequent LHC revealed a 90% complex, stenotic, fibro-fatty lesion extending from the proximal LAD artery to the mid-LAD artery (Figures 2, 3). Intravascular ultrasound (IVUS) confirmed a severe plaque burden. Additional findings included a 100% thrombotic occlusion of the first diagonal (D1) branch (Figure 4), a 60% stenotic tubular lesion from the ostial to proximal right coronary artery (RCA), and elevated left ventricular end-diastolic pressure (LVEDP) at 18 mmHg.

**Figure 5:**
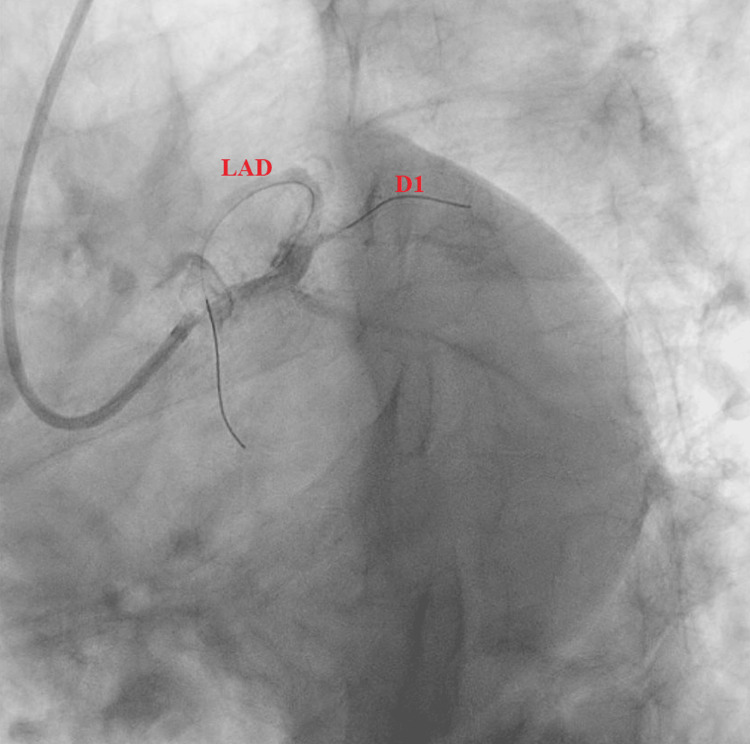
Left coronary angiogram demonstrating guidewires successfully advanced into the left anterior descending (LAD) and first diagonal (D1) arteries prior to stent deployment

**Figure 6:**
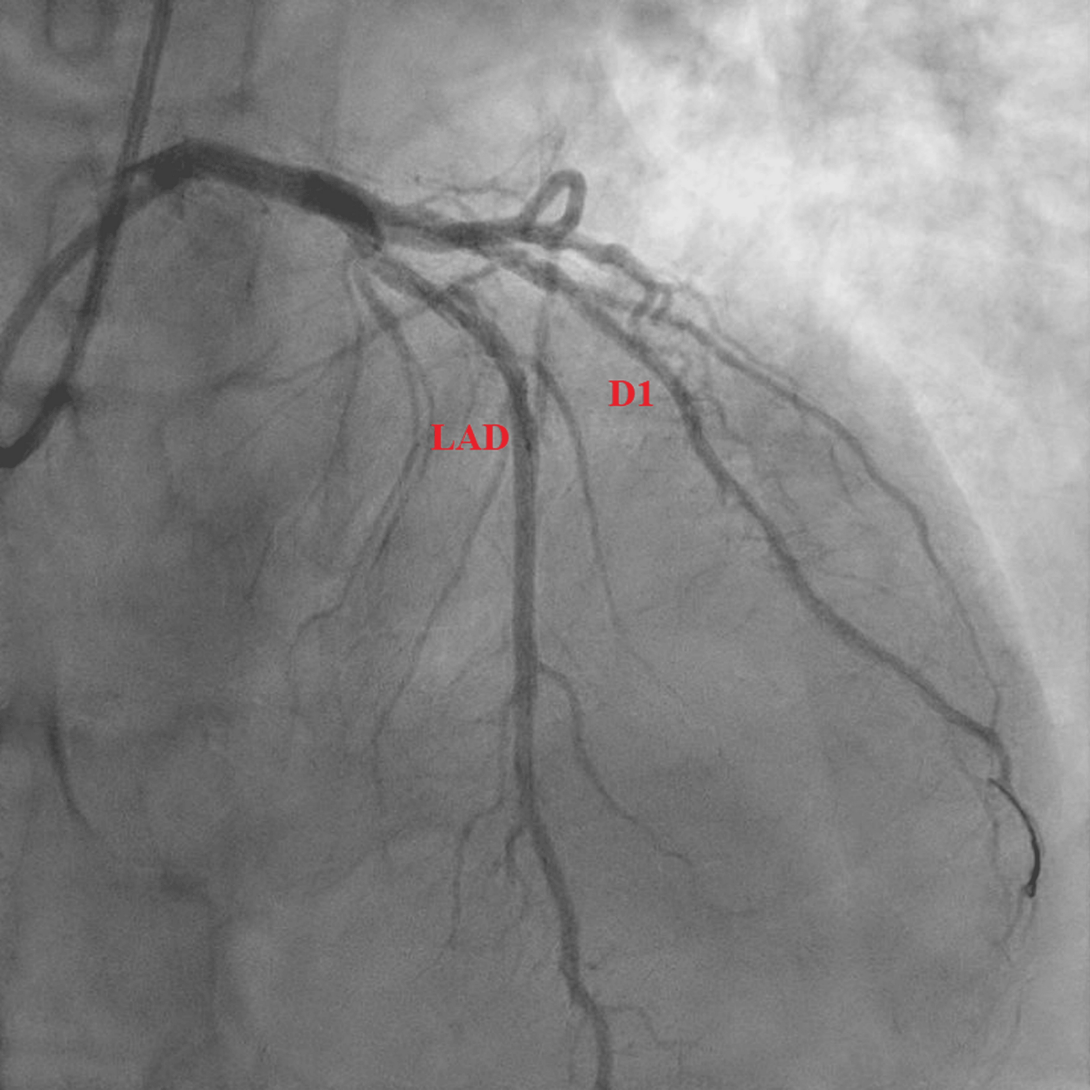
Left coronary angiogram following successful percutaneous coronary intervention with stent placement in the left anterior descending (LAD) artery and the first diagonal (D1) branch, showing restoration of flow in both vessels

The patient underwent PCI to the culprit vessel (D1), which was successfully stented with a 2.5 × 15 mm Resolute drug-eluting stent. Advancement of the guidewire was challenging, suggesting probable subacute closure of the vessel. PCI was also performed on the LAD lesion using a 3.5 × 30 mm Resolute stent (Figures 5, 6). Post-stent expansion and apposition were confirmed by IVUS, with a minimum luminal area (MLA) greater than 8.5 mm². The patient was monitored in the step-down unit without complications. Dual antiplatelet therapy with aspirin and ticagrelor was initiated in addition to high-intensity statin therapy.

**Figure 7:**
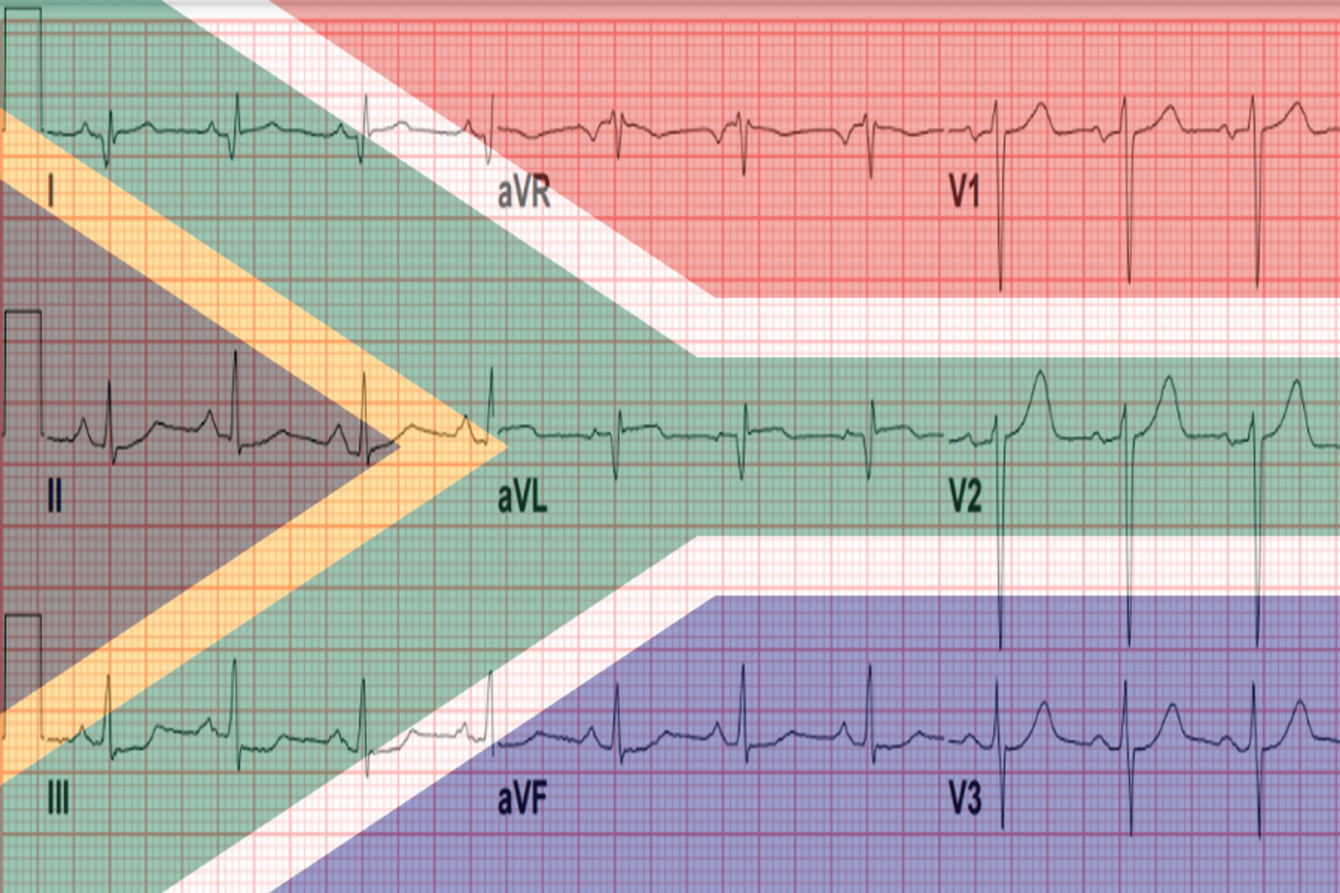
The patient's ECG demonstrating the South African flag sign (SAFS), characterized by subtle ST-segment elevation in leads I, aVL, and V2 with reciprocal ST-segment depression in lead III. The background overlay of the South African national flag highlights the characteristic “Y-shaped” pattern formed by these leads, from which the sign derives its name. The national flag of South Africa, illustrating the visual analogy underlying the South African flag sign on ECG, is republished from Wikimedia Commons [7] and licensed under CC BY-SA 3.0 (https://creativecommons.org/licenses/by-sa/4.0/). No additional modifications were made beyond educational and illustrative overlay.

**Figure 8:**
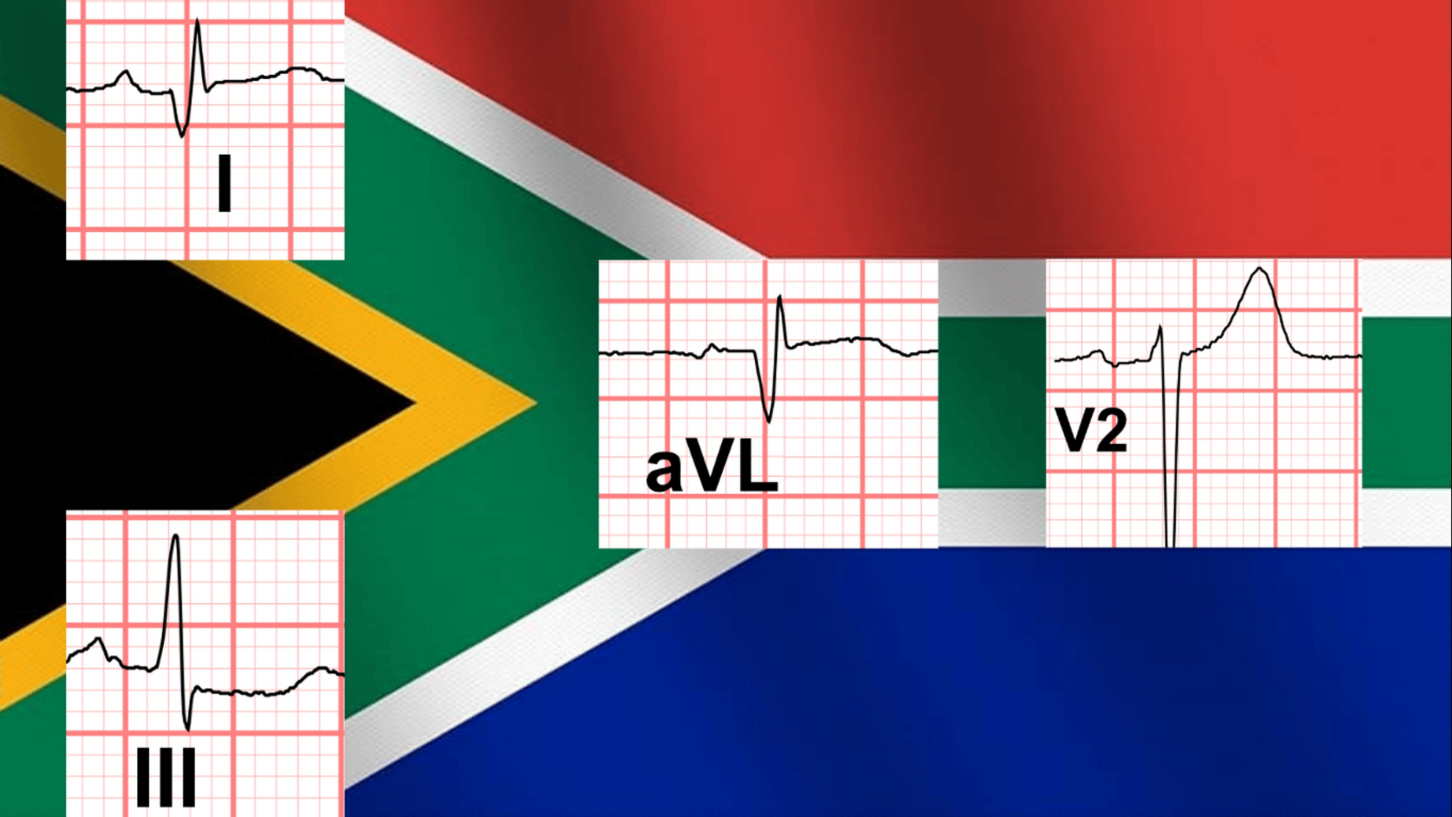
The patient's ECG leads of interest illustrating the South African flag sign (SAFS). The national flag of South Africa, illustrating the visual analogy underlying the South African flag sign on ECG, is republished from Wikimedia Commons [7] and licensed under CC BY-SA 3.0 (https://creativecommons.org/licenses/by-sa/4.0/). No additional modifications were made beyond educational and illustrative overlay.

**Figure 9:**
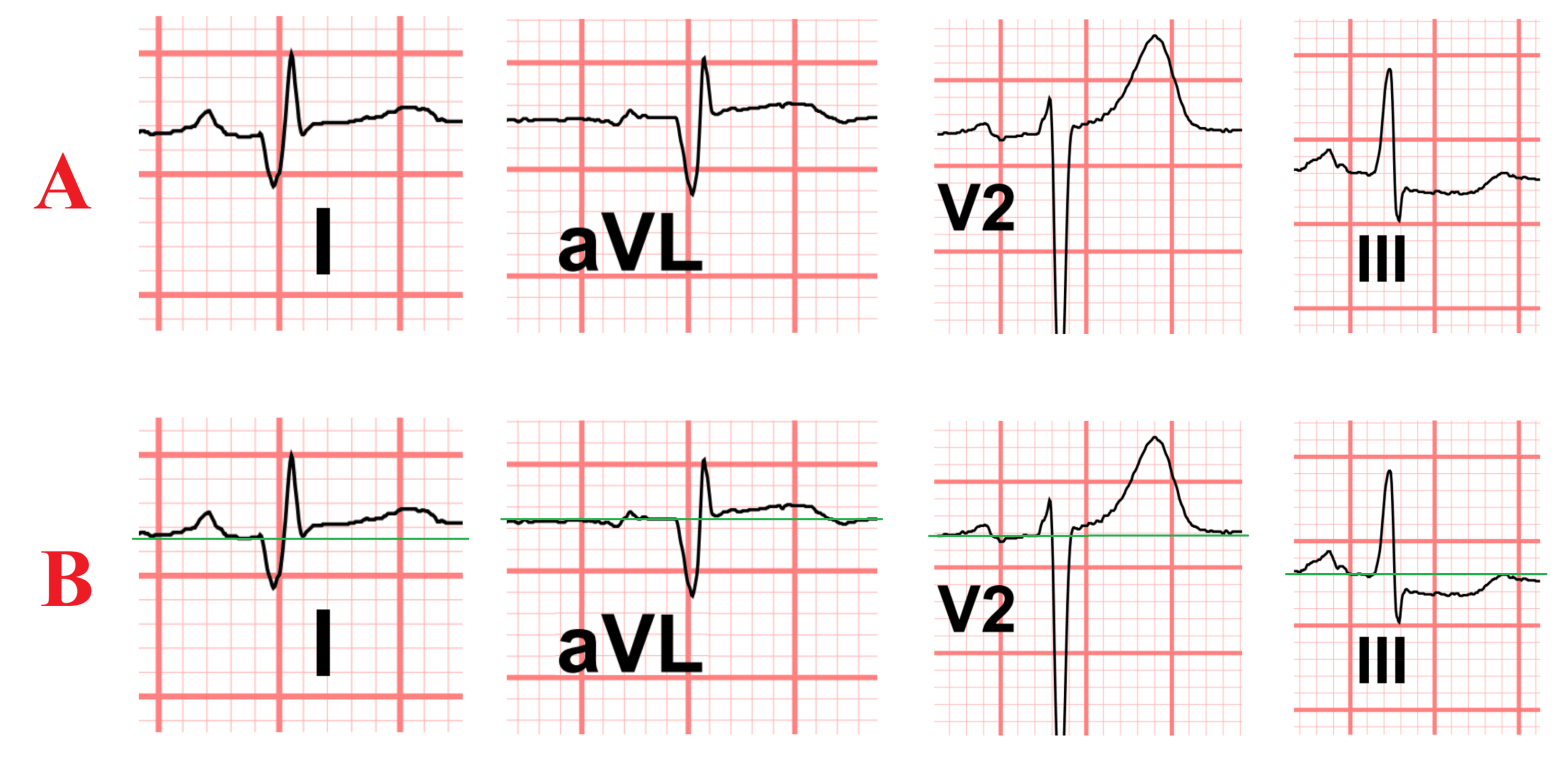
Close-up view of the patient's ECG leads demonstrating the South African flag sign (SAFS) (A) Leads I, aVL, V2, and III, illustrating the characteristic pattern of ST-segment elevation in I, aVL, and V2, with reciprocal ST-segment depression in III. (B) The same leads with a green reference line drawn along the isoelectric baseline to highlight subtle ST-segment deviations more clearly.

Retrospective review of the initial ECG revealed a subtle SAFS, with minimal ST-segment elevation in leads I, aVL, and V2, and reciprocal ST-segment depression in lead III (Figures 7, 8, 9 (Panels A and B)). This finding was consistent with total occlusion of the first diagonal artery.

## Discussion

The SAFS represents a distinctive yet underrecognized electrocardiographic (ECG) pattern associated with occlusion of the first diagonal branch (D1) of the LAD artery. First described by Dr. László Littmann in 2016 in The American Journal of Emergency Medicine [8], the sign derives its name from the alignment of ST-segment deviations that mirror the Y-shaped geometry and color alignment of the South African flag. It is defined by ST-segment elevation in leads I, aVL, and V2, with reciprocal ST depression in lead III, reflecting ischemia in the high anterolateral myocardial territory supplied by the D1 branch. Because these leads are noncontiguous on the standard 12-lead ECG layout, the pattern can be subtle and easily misclassified as NSTEMI, despite representing a true ACO.

Careful ECG interpretation is crucial in identifying the SAFS and differentiating it from other electrocardiographic patterns. The SAFS can be differentiated from other causes of ST-segment elevation by its distinctive noncontiguous lead involvement (I, aVL, and V2) with reciprocal ST-segment depression in lead III. In contrast, early repolarization typically demonstrates diffuse, concave ST elevation without reciprocal changes; pericarditis presents with widespread ST elevation and PR depression; LV hypertrophy meets voltage criteria with a strain pattern often limited to lateral leads; and Takotsubo cardiomyopathy usually generates anterior ST elevation extending beyond V2 without reciprocal changes. The presence of reciprocal ST depression and localization to the diagonal artery territory makes SAFS highly specific for ACO rather than non-ischemic etiologies.

The SAFS is not merely an academic curiosity. It represents an ECG footprint of ACO of a diagonal branch, which, if left unrecognized, carries the same risks as “classic” STEMI lesions, including myocardial necrosis, heart failure, arrhythmias, and death. In published case reports, patients demonstrating SAFS often had elevated biomarkers, LV dysfunction, or regional wall motion abnormalities. One review of SAFS noted that prompt PCI in two out of four SAFS cases led to favorable outcomes, while delayed recognition may have led to larger infarcts [9]. Because diagonal branches supply portions of the lateral and anterior wall, even a small territory of occlusion can produce significant morbidity if timely reperfusion is not achieved. Thus, when SAFS is identified, it warrants the same urgency as overt STEMI.

Historically, the management of acute myocardial infarction (AMI) has been guided by the STEMI/NSTEMI dichotomy - a framework that assumes the presence of ST elevation as a surrogate for an ACO. However, emerging evidence challenges this assumption. In a pivotal study by Meyers et al. (2021), blinded interpretation using predefined OMI-based ECG findings demonstrated more than double the sensitivity (86% vs 41%) of the STEMI criteria for diagnosing acute occlusions, while maintaining comparable specificity [2]. Similarly, Kola et al. (2024) showed that 40% of patients with angiographically confirmed OMI did not meet STEMI criteria, leading to significant delays in catheterization and consequently higher morbidity and mortality [1]. These findings emphasize that the presence or absence of coronary occlusion, rather than arbitrary millimeter-based ST elevation, should dictate reperfusion strategies.

The OMI/NOMI paradigm, first conceptualized by Smith, Meyers, and Aslanger in their OMI Manifesto (2018), reframes acute MI classification around the actual pathophysiology - the presence of an ACO. Under this model, the SAFS serves as a quintessential example of an OMI pattern that falls outside the classical STEMI criteria but carries equivalent clinical urgency. As McLaren et al. (2024) emphasized, the STEMI paradigm “misses a significant minority of patients with acute coronary occlusion,” with an estimated 25%-30% of NSTEMI patients harboring unrecognized total occlusions, often resulting in delayed reperfusion and higher mortality [6]. The OMI framework, therefore, encourages clinicians to integrate advanced ECG interpretation, bedside echocardiography, and high-sensitivity biomarkers to identify occlusions early, regardless of ST-segment elevation magnitude.

Across published literature, the SAFS has consistently emerged as a subtle but diagnostically powerful ECG marker of first diagonal (D1) artery occlusion. Several case reports mirror our patient’s presentation, such as transient or minimal chest pain symptoms, mild ST-segment changes, and an initial NSTEMI classification, followed by angiographic confirmation of D1 occlusion. Rajendran et al. (2021) described a 58-year-old man with retrosternal pain and the classic SAFS pattern - ST elevation in leads I, aVL, V2, and reciprocal depression in III - who was initially misdiagnosed with lateral wall ischemia before catheterization confirmed D1 occlusion [3]. They emphasized that noncontiguous ST-segment elevation in I/aVL and V2 is easily missed under the STEMI/NSTEMI framework and highlighted Littmann (2016) as the first to coin the “South African Flag Sign” as an ECG teaching tool for high-lateral infarcts.

In a related case, Naranjo and Hermosillo (2023) reported an 80-year-old diabetic woman whose ECG displayed complete right bundle-branch block with ST elevation in I, aVL, V2, and ST depression in III - the classic SAFS configuration. Her angiogram revealed thrombotic D1 occlusion with TIMI-2 flow, and although stenting was not feasible due to vessel caliber < 2 mm, the case reaffirmed that the SAFS localizes ischemia to the high-lateral wall and mandates prompt recognition to prevent missed infarctions [10].

More recently, Luckmann et al. (March 2025) presented a four-patient case series confirming the reproducibility of this ECG signature. Two patients had acute D1 occlusion successfully treated with PCI, whereas two exhibited the SAFS pattern without obstructive lesions, showing its high sensitivity but limited specificity. The authors argued that SAFS should trigger urgent invasive evaluation equivalent to new left bundle branch block (LBBB) in ACS, since many cases otherwise labeled as NSTEMI indeed involve an occlusive culprit lesion requiring immediate reperfusion [9].

Expanding the anatomic spectrum, Swarath et al. (2023) described a 47-year-old woman whose SAFS corresponded not to D1 occlusion but to a 100% proximal LAD (“widow-maker”) lesion. This finding underscores that the SAFS can occasionally herald other occlusive territories, reinforcing that clinicians should pursue an OMI-based strategy that emphasizes the presence of arterial occlusion over ST millimeter criteria [6].

Taken together, these reports share key themes: subtle ECG changes misclassified as NSTEMI, angiographic confirmation of occlusion (most often D1), and the importance of rapid PCI once the SAFS is recognized. Our patient’s presentation - with mild chest pain, fatigue, and minimal ST-segment deviation but a proven 100% thrombotic D1 occlusion successfully stented - closely parallels the diagonal-culprit cases described by Naranjo and Hermosillo [10] and Luckmann et al. [9], reinforcing the reliability of SAFS as a marker of OMI even when standard STEMI thresholds are not met.

The SAFS thus embodies the essence of the OMI paradigm: subtle ECG findings indicating acute vessel occlusion in the absence of classical STEMI features. Under the conventional STEMI/NSTEMI classification, our patient was categorized as NSTEMI, and, as a result, coronary angiography was deferred until the following morning. This delay ultimately revealed a D1 thrombotic occlusion, emphasizing how strict adherence to STEMI criteria can lead to under-recognition of true occlusive events. Had the OMI perspective been applied - recognizing that the key determinant of outcome is the presence of coronary occlusion rather than the fulfillment of ST-elevation thresholds - earlier intervention might have been achieved (Table 2). This case highlights the critical importance of careful ECG interpretation, not only in identifying classic STEMI patterns but also in detecting STEMI equivalents such as SAFS, which can guide timely reperfusion and improve patient outcomes.

**Table 2:** Comparison of the STEMI/NSTEMI and OMI/NOMI paradigms as applied to our patient’s case, highlighting differences in ECG interpretation, diagnostic sensitivity, clinical management, and outcomes OMI: Occlusion myocardial infarction; NOMI: Non-occlusion myocardial infarction; PCI: Percutaneous coronary intervention; STE: ST-segment elevation; STD: ST-segment depression; STEMI: ST-elevation myocardial infarction; NSTEMI: Non-ST-elevation myocardial infarction; LHC: Left heart catheterization.

Parameter	STEMI/NSTEMI Interpretation	OMI/NOMI Interpretation	Patient Case Findings
ECG criteria	No diagnostic ST elevation (does not meet STEMI criteria); labeled as NSTEMI	South African flag sign identified (STE in I, aVL, and V2 with reciprocal STD in III) consistent with OMI	Subtle STE in I, aVL, V2; STD in III
Diagnostic sensitivity for occlusion	Missed total occlusion because STEMI criteria were not met	Correctly flagged as OMI despite not fulfilling STEMI cutoffs	100% D1 thrombotic occlusion confirmed on LHC
Clinical pathway	Typically leads to early/delayed invasive strategy (NSTEMI workup)	Supports urgent reperfusion as in STEMI equivalent	Catheterization revealed D1 occlusion; PCI was performed
Outcome relevance	Risk of under-recognition and delayed reperfusion	More accurate identification of the culprit vessel and timely PCI	Delayed PCI resulted in an uneventful recovery

Within the broader OMI/NOMI paradigm, these findings demonstrate why traditional STEMI/NSTEMI classification can fail to capture high-risk occlusions. As advocated by Sankardas et al. in 2021, reclassifying infarctions around the presence or absence of occlusion - rather than arbitrary ST-segment magnitude - improves diagnostic accuracy and ensures timely reperfusion [11]. The SAFS thus epitomizes the OMI principle: a subtle, often overlooked ECG sign that signals a complete coronary obstruction requiring emergent intervention. By integrating this paradigm into clinical practice, clinicians can avoid diagnostic inertia in STEMI-negative but occlusion-positive cases like ours, improving outcomes through earlier angiography and PCI. This convergence of case evidence positions the SAFS not merely as a descriptive ECG curiosity but as a clinically actionable OMI pattern warranting the same urgency as classical STEMI.

Emerging literature advocates for a shift in clinical algorithms toward OMI-based identification and management. The inclusion of SAFS, de Winter, Wellens’, and other nontraditional OMI equivalents into acute MI protocols may improve recognition of critical lesions that evade standard STEMI criteria. Artificial intelligence-assisted ECG analysis and machine learning algorithms, as suggested by McLaren et al. (2024) [6], may further enhance early detection of OMI by integrating spatial and temporal ECG patterns that human readers often miss. Ultimately, broad adoption of the OMI/NOMI paradigm may reduce treatment delays, improve revascularization timing, and lower mortality in patients with subtle yet life-threatening coronary occlusions.

## Conclusions

The SAFS represents a subtle yet clinically significant electrocardiographic marker of acute diagonal branch occlusion that may be overlooked under the traditional STEMI/NSTEMI paradigm, particularly when ST-segment elevation in leads I and aVL is minimal. In our case, the absence of diagnostic ST elevation by classical criteria resulted in delayed coronary angiography until the following morning, at which point a 100% thrombotic occlusion of the first diagonal (D1) artery was identified. This case is unique in demonstrating angiographically confirmed total D1 occlusion despite not meeting STEMI thresholds, emphasizing the limitations of millimeter-based STEMI criteria and highlighting the SAFS as an early indicator of OMI. It also reinforces the clinical value of the OMI paradigm, which prioritizes the identification of acute vessel occlusion over rigid ECG cutoffs and supports timely reperfusion to improve patient outcomes.
